# Comparison of sonographic fetal weight estimation formulas in patients with preterm premature rupture of membranes

**DOI:** 10.1186/s12884-021-03631-w

**Published:** 2021-02-19

**Authors:** Chelsie Warshafsky, Stefania Ronzoni, Paula Quaglietta, Eran Weiner, Arthur Zaltz, Jon Barrett, Nir Melamed, Amir Aviram

**Affiliations:** 1 Sunnybrook Health Sciences Centre, Department of Obstetrics and Gynecology, Division of Maternal-Fetal Medicine, University of Toronto, 2075 Bayview Ave, Toronto, ON M4N 3M5 Canada; 2 Edith Wolfson Medical Center, Department of Obstetrics and Gynecology, Division of Maternal-Fetal Medicine, Holon, Sackler Faculty of Medicine, Tel-Aviv University, Tel-Aviv, Israel

**Keywords:** Estimation of fetal weight, Ultrasound, Preterm premature rupture of the membranes, Hadlock IV

## Abstract

**Background:**

Estimation of fetal weight (EFW) by ultrasound is useful in clinical decision-making. Numerous formulas for EFW have been published but have not been validated in pregnancies complicated by preterm premature rupture of membranes (PPROM). The purpose of this study is to compare the accuracy of EFW formulas in patients with PPROM, and to further evaluate the performance of the most commonly used formula - Hadlock IV.

**Methods:**

A retrospective cohort study of women with singleton gestations and PPROM, admitted to a single tertiary center between 2005 and 2017 from 22^0/7^–33^0/7^ (*n* = 565). All women had an EFW within 14 days of delivery by standard biometry (biparietal diameter, head circumference, abdominal circumference and femur length). The accuracy of previously published 21 estimated EFW formulas was assessed by comparing the Pearson correlation with actual birth weight, and calculating the random error, systematic error, proportion of estimates within 10% of birth weight, and Euclidean distance.

**Results:**

The mean gestational was 26.8 ± 2.4 weeks at admission, and 28.2 ± 2.6 weeks at delivery. Most formulas were strongly correlated with actual birth weight (r > 0.9 for 19/21 formulas). Mean systematic error was − 4.30% and mean random error was 14.5%. The highest performing formula, by the highest proportion of estimates and lowest Euclidean distance was Ott (1986), which uses abdominal and head circumferences, and femur length. However, there were minimal difference with all of the first 10 ranking formulas. The Pearson correlation coefficient for the Hadlock IV formula was strong at r = 0.935 (*p* < 0.001), with 319 (56.5%) of measurements falling within 10%, 408 (72.2%) within 15% and 455 (80.5%) within 20% of actual birth weight. This correlation was unaffected by gender (r = 0.936 for males, r = 0.932 for females, *p* < 0.001 for both) or by amniotic fluid level (r = 0.935 for mean vertical pocket < 2 cm, r = 0.943 for mean vertical pocket ≥2 cm, *p* < 0.001 for both).

**Conclusions:**

In women with singleton gestation and PPROM, the Ott (1986) formula for EFW was the most accurate, yet all of the top ten ranking formulas performed quite well. The commonly used Hadlock IV performed quite similarly to Ott’s formula, and is acceptable to use in this specific setting.

**Supplementary Information:**

The online version contains supplementary material available at 10.1186/s12884-021-03631-w.

## Background

Ultrasound is one of the most useful tools for antepartum fetal surveillance. Estimation of fetal weight (EFW) by ultrasound is a key component in antepartum monitoring and management, as a means of assessing fetal growth and overall wellbeing. Yet, since ultrasound became one of the mainstays of obstetrical care, multiple formulas for calculation of EFW have been proposed [1–12]. These formulas use a variation of fetal biometrics, including abdominal circumference (AC), femur length (FL), head circumference (HC), and biparietal diameter (BPD). Many studies have been performed to assess the accuracy of these formulas in specific scenarios including small for gestational age [13], large for gestational age [14, 15], macrosomia [16], fetal gender [17, 18], and multiple gestation [19, 20].

Preterm premature rupture of membranes (PPROM) affects 2–3% of pregnancies [21, 22]. While no one uniform antenatal monitoring protocol exists, management usually includes initial admission to an obstetrics unit, antibiotic therapy, daily vital signs assessment, and periodic non-stress tests (NST), with or without ultrasound investigations as well [22]. The decision regarding timing and mode of delivery is one based on clinical judgement combining all of these parameters. Ultrasound evaluation and imaging are affected by multiple factors, including the level of amniotic fluid [23]. Whether the same EFW formulas used for general antepartum purposes can also be used in the setting of PPROM is a matter of debate, and previous studies were either limited by sample size or focused on one formula only. Therefore, the primary objective of this study was to evaluate the accuracy of the various sonographic EFW formulas in patients with PPROM.

## Methods

### Study design and participants

This was a retrospective study of women with singleton gestations admitted with PPROM between 22 + 0/7 and 33 + 6/7 weeks of gestation to a university-affiliated, tertiary hospital between January 2005 and December 2017. Exclusion criteria included those with acute chorioamnionitis necessitating immediate delivery, and pregnancies with major fetal genetic or structural anomalies [24]. The study was approved by the local institutional review board (approval #145–2018).

Gestational age was determined using the last menstrual period and was confirmed by a first trimester sonogram [24]. Rupture of membranes was determined based on clinical history and at least one supporting physical examination finding (significant pooling of fluid in the posterior fornix, positive ferning test, or positive AmniSure test). As per the departmental protocol, women with confirmed PPROM were admitted to the antepartum obstetrical unit until delivery [24]. They received antibiotics, antenatal corticosteroids, and were monitored for signs of intrauterine infection. Vital signs were taken four times daily. Fetal heart rate auscultation was performed twice a day, in addition to a daily NST. Also, in accordance with our protocol, all admitted women with PPROM had a twice weekly ultrasound for biophysical profile (BPP) and umbilical artery pulsatility index (UA PI), as an adjunct measure for fetal well-being. An EFW was performed at admission, and every 14 days until delivery [24].

### Test methods and analysis

The accuracy of twenty-one previously published EFW formulas was tested using the following measures:

1. Pearson correlation coefficient (r), which represent the correlation between the EFW and the actual birth weight (BW). The closer R is to 1, the greater the linear correlation between the EFW and BW.
2. Systematic error [SE = (EFW-BW)/BW*100], which reflects the inherent systematic deviation of a given formula from the actual BW.
3. Random error (RE, calculated as the standard deviation of the SE), which reflects the random component of prediction error.
4. Euclidean distance $=\left(\sqrt[2]{SE^2+{RE}^2}\right)$, which represents the geometric average of the SE and RE, expressed as percentages. The smaller the SE and RE are, the smaller the Euclidean distance and thus the more accurate the formula is. This measure was also used to determine the overall ranking of the formulas.
5. Proportion of estimates (POE) within ±10% of birth weight, which reflects the percentage of measurements within + 10% and − 10% of the actual BW.
6. For small-for-gestational age (SGA, birthweight<10th percentile for gestational age), we calculated the percentage of neonates who were correctly identified by each formula as SGA (Identified as SGA by formula / SGA * 100). Birth weight percentiles were determined based on local population growth curves [25].

The primary objective of this study was to evaluate the accuracy of the various sonographic EFW formulas in patients with PPROM. The secondary objective was to further evaluate the performance of the Hadlock IV formula, as it is one of the most commonly formulas used to today.

Statistical analysis was done using the SPSS software (version 25.0, IBM software, Armnok, NY, USA).

## Results

### Participants

There were 45,943 singleton deliveries at our institution during the study period. Of those, 845 had PPROM prior to 34 weeks of gestation, and 565 women met the inclusion criteria. Demographic data is presented in Table 1. Mean gestational age at admission was 26.8 ± 2.4 weeks, mean gestational age at delivery was 28.2 ± 2.6 weeks, and the median time from EFW to delivery was 3 days (Table 1). Of note, 443 (78%) of the sonographic EFW were performed within 7 days of delivery.

**Table 1:** Demographic data

Variable	
Maternal age, years (mean ± SD)	32.2 ± 5.5
Parity, n (median [range])	1 [0–9]
Gestational age at admission, weeks (mean ± SD)	26.8 ± 2.4
Gestational age at delivery, weeks (mean ± SD)	28.2 ± 2.6
Sonogram to delivery interval, days (median [range])	3 [0–14]
Birth weight, grams (mean ± SD)	1154 ± 418
Oligohydramnios (MVP < 2 cm), n(%)	279 (49.4)
Male neonates, n(%)	317 (56.1)

*SD* Standard deviation, *MVP* Mean vertical pocket

### Test results

The twenty-one formulas are listed in Table 2, and the comparison between their performance is presented in Table 3. Most formulas had a strong correlation with actual birth weight (19/21 formulas with r > 0.9). The strongest correlation was calculated for formulas using both FL and HC in addition to AC, such as Combs [11] (r = 0.940), Ott [12] (*r* = 0.940), and Hadlock [1] (*r* = 0.936).

**Table 2:** Common formulas used for sonographic fetal weight estimation

Group 1 (AC and FL)		
1	Hadlock et al. (1985) [1]	=10 ^1.304 + 0.05281(AC) + 0.1938(FL)-0.004(AC)(FL)^
2	Woo et al. (1985) [2]	=10 ^0.59 + 0.08(AC) + 0.28(FL)-0.00716(AC)(FL)^
3	Warsof et al. (1986)^a^ [3]	=e^2.792 + 0.108(FL) + 0.0036(AC)2–0.0027(FL)(AC)^
Group 2 (AC and BPD)		
4	Vintzileos et al. (1987) [4]	=10 ^1.879 + 0.0084(BPD) + 0.026(AC)^
5	Warsof et al. (1977)^b^ [5]	=10 ^− 1.599 + 0.144(BPD) + 0.032(AC) − 0.000111(BPD)2(AC)^
6	Shepard et al. (1982)^b^ [6]	=10^–1.7492 + 0.166(BPD) + 0.046(AC)-0.002546(AC)(BPD)^
7	Jordaan (1983)^b^ [7]	=10^–1.1683 + 0.0377(AC) + 0.0950(BPD)-0.0015(BPD)(AC)^
8	Hadlock et al. (1984) [8]	=10 ^1.1134 + 0.05845(AC) − 0.000604(AC)2–0.007365 (BPD)2 + 0.000595(BPD)(AC) + 0.1694(BPD)^
9	Woo et al. (1985) [2]	=10 ^1.63 + 0.16(BPD) + 0.00111(AC)2–0.0000859 (BPD)(AC)2^
10	Hsieh at al. (1987) [9]	=10 ^2.1315 + 0.0056541(AC)(BPD) − 0.00015515 (BPD)(AC)2 + 0.000019782(AC)3 + 0.052594(BPD)^
Group 3 (AC and HC (±BPD))		
11	Hadlock et al. (1984) [8]	=10 ^1.182 + 0.0273(HC) + 0.07057(AC) − 0.00063 (AC)2–0.0002184(HC)(AC)^
12	Jordaan (1983) [7]	=10 ^0.9119 + 0.488(HC) + 0.0824(AC)-0.001599(HC)(AC)^
13	Jordaan (1983) [7]	=10 ^2.3231 + 0.02904(AC) + 0.0079(HC) − 0.0058(BPD)^
Group 4 (AC, FL and BPD)		
14	Hadlock et al. (1985) [1]	=10 ^1.335–0.0034(AC)(FL) + 0.0316(BPD) + 0.0457(AC) + 0.1623(FL)^
15	Woo et al. (1985) [2]	=10 ^1.54 + 0.15(BPD) + 0.00111(AC)2–0.0000764(BPD)(AC)2 + 0.05(FL) − 0.000992(FL)(AC)^
16	Shinozuka et al. (1987) [10]	= 0.23966(AC)^2^(FL) + 1.6230(BPD)^3^
17	Hsieh at al. (1987) [9]	=10 ^2.7193 + 0.0094962(AC)(BPD) − 0.1432(FL) − 0.00076742(AC)(BPD)2 + 0.001745(FL)(BPD)2^
Group 5 (AC, FL and HC)		
18	Hadlock et al. (1985) [1]	=10 ^1.326–0.00326(AC)(FL) + 0.0107(HC) + 0.0438(AC) + 0.158(FL)^
19	Combs et al. (1993) [11]	=0.23718(AC)^2^ (FL) + 0.03312(HC)^3^
20	Ott et al. (1986)^b^ [12]	=10 ^− 2.0661 + 0.04355(HC) + 0.05394(AC) − 0.0008582(HC)(AC) + 1.2594(FL/AC)^
Group 6 (AC, FL, BPD and HC)		
21	Hadlock et al. (1985) [1]	=10 ^1.3596 + 0.0064(HC) + 0.0424(AC) + 0.174(FL) + 0.00061(BPD)(AC) − 0.00386(AC)(FL)^

AC Abdominal circumference, BPD Biparietal diameter, FL Femur diaphysis length and HC Head circumference expressed in cm and estimated fetal weight (EFW) expressed in grams, unless stated otherwise. ^a^FL expressed in mm. ^b^EFW expressed in kg

**Table 3:** Comparison of twenty-one formulas for fetal weight estimation

Formula #	Reference	Pearson coefficient (r)	SE (%)	RE (%)	POE < 10% (%)	ED (%)	Identified as SGA (%)	Rank
**Group 1 (AC and FL)**								
1	Hadlock 1 (1985)	0.928	−2.35	13.27	58.6	13.48	48.8	8
2	Woo (1985)	0.929	−7.98	14.91	44.4	16.91	58.5	14
3	Warsof (1986)	0.913	2.44	15.41	50.6	15.60	41.5	12
**Group 2 (AC and BPD)**								
4	Vintzileos (1987)	0.923	−0.76	14.08	54.3	14.10	31.7	10
5	Warsof (1977)	0.922	−13.42	12.06	32.6	18.04	63.4	15
6	Shepard (1982)	0.924	− 4.90	13.25	54.0	14.13	46.3	11
7	Jordaan (1983)	0.928	14.83	17.21	36.6	22.72	12.2	19
8	Hadlock (1984)	0.925	−4.34	13.17	56.5	13.87	46.3	9
9	Woo (1985)	0.922	−13.66	11.99	30.1	18.18	63.4	16
10	Hsieh (1987)	0.754	−80.15	21.50	0.7	82.98	90.2	21
**Group 3 (AC and HC (±BPD))**								
11	Hadlock (1984)	0.937	−4.69	12.26	56.8	13.13	46.3	3
12	Jordaan (1983)	0.930	13.29	15.17	37.7	20.17	19.5	18
13	Jordaan (1983)	0.927	28.11	24.00	20.2	36.96	4.9	20
**Group 4 (AC, FL and BPD)**								
14	Hadlock 2 (1985)	0.933	−4.90	12.39	57.7	13.32	48.8	7
15	Woo (1985)	0.925	−10.66	12.24	41.8	16.23	51.2	13
16	Shinozuka (1987)	0.932	0.62	13.09	60.9	13.10	34.1	2
17	Hsieh (1987)	0.895	5.14	18.40	46.4	19.10	14.6	17
**Group 5 (AC, FL and HC)**								
18	Hadlock 3 (1985)	0.936	−5.17	12.16	57.3	13.21	48.8	4
19	Combs (1993)	0.940	3.23	12.85	58.9	13.25	29.3	5
20	Ott (1986)	0.940	0.36	12.42	62.8	12.43	71.4	1
**Group 6 (AC, FL, BPD and HC)**								
21	Hadlock 4 (1985)	0.935	−5.40	12.17	56.6	13.31	48.8	6

*SE* Systemic error, *RE* Random error, *POE* Proportion of estimations, *ED* Euclidean distance, *SGA* Small for gestational age, *AC* Abdominal circumference, *FL* Femur length, *BPD* Biparietal diameter, *HC* Head circumference

**Fig. 1:**
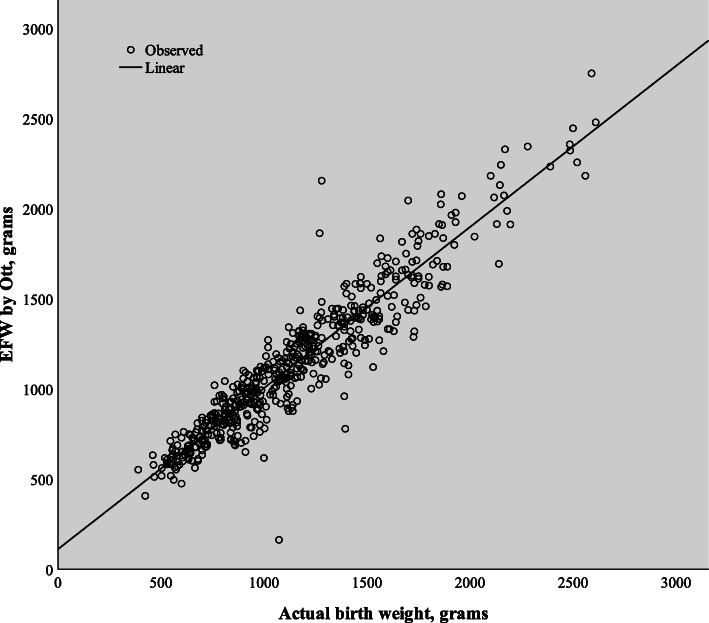
Correlation between sonographic estimated fetal weight and actual birth weight for the Ott formula (*r* = 0.884)

The SE ranged from 0.36% (Ott [12]) to − 80.15% (Hsieh [9]), with a mean of − 4.30%. The RE ranged from 11.99% (Woo [2]) to 24.00% (Jordaan [7]) with a mean of 14.48%. The largest POE < 10% was achieved by the Ott [12] formula, with 62.8% of EFW within ±10% of the actual BW. The formula that was the most accurate, by the lowest Euclidean distance, was again that of Ott [12], utilizing AC, FL and HC (Fig. 1).

Yet, the Euclidean distance of the top 10 ranking formulas differed only slightly from one to another, ranging from 12.43 to 14.10%, indicating they all performed quite similarly. The formula by Hsieh [9] accurately detected the largest percentage of SGA neonates (90.2%), followed by the Ott’s formula (71.4%) [12], then the Hadlock IV formula [1]. We also examined the difference between the performance of the formulas when EFW was done between 1 and 7 days prior to delivery, and 8–14 days prior to delivery. As expected, for the vast majority of formulas (17/21 formulas), the POE within 10% of actual birth weight was higher for EFW performed closer to delivery (Supplementary Table S1).

**Fig. 2:**
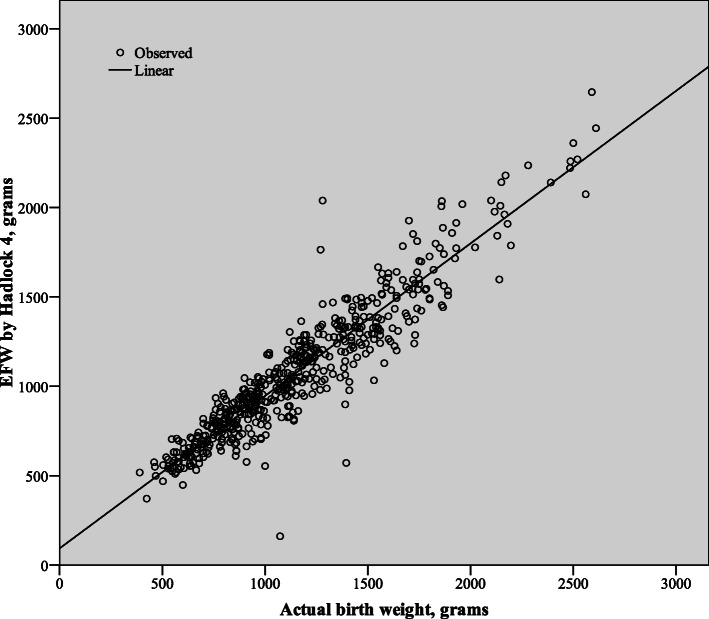
Correlation between sonographic estimated fetal weight and actual birth weight for the Hadlock IV formula (*r* = 0.935)

The Hadlock IV formula [1] is commonly used in clinical practice and is integrated in most commercially used ultrasound machines. Thus, we chose to analyze its performance in further detail. The Pearson correlation coefficient for the Hadlock IV formula was strong (*r* = 0.935, Fig. 2). This correlation was not affected by gender (*r* = 0.932 for females, *r* = 0.936 for males) or by level of amniotic fluid (*r* = 0.935 for MVP < 2 cm, *r* = 0.943 for MVP ≥ 2 cm). A total of 56.5% of EFW fell within the ±10% range of the actual BW, with 72.2% of EFW within the ±15% of the actual BW and 80.5% of EFW within the ±20% margins of the actual BW.

## Discussion

In this study, we aimed to evaluate the performance of previously published EFW formulas in pregnancies complicated by PPROM.

We have found that Ott’s formula [12] is the most accurate formula, based on the smallest Euclidean Distance and largest POE < 10%. Yet, the differences in performance between most formulas, especially the top 10 ranking formulas, were quite negligible. We also found that the commonly used Hadlock IV formula [1] was accurate, with more than 70% of estimations falling between ±15% of the actual BW, and performs well in the setting of PPROM, regardless of fetal gender or amniotic fluid volume.

One of the earliest studies investigating EFW in pregnancies complicated by PPROM was conducted in 1990 by Valea et al. [26]. They compared the accuracy of EFW in low birth weight fetuses, reviewing 86 fetuses with PPROM and 112 fetuses with intact membranes using two different EFW formulas: Shepard [6] which uses BPD and AC, and Rose and McCallum [27], which uses BPD, FL, and mean abdominal diameter. They determined both formulas were equally applicable regardless of membrane status. Toohey [28] and colleagues performed a similar study in 1991, assessing 98 patients with PPROM who had EFW and amniotic fluid index assessments within 48 h of delivery, compared to 55 patients in preterm labour with intact membranes and normal amniotic fluid indices. Using the Shepard [6] and Hadlock [1] formulas they found that EFW in PPROM is as accurate as those with intact membranes. These studies were both done almost 30 years ago and had small sample sizes. Their accuracy is affected both by the ultrasound machines that were used at that time, and by the fact that very preterm fetuses were not included.

A more contemporary study was performed by Esin et al. [29] and found results similar to ours. Nonetheless, the sample size of this study was half of ours, and the mean gestational age at delivery in their study was almost 5 weeks later than ours (28.2 ± 2.6 weeks in our study vs. 32.4 ± 3.2 weeks in Esin et al.), affecting the accuracy of the formulas. In contrast, Duncan et al. [23] recently published their prospective cohort assessing the accuracy of the Hadlock IV formula in patients with PPROM. They found that EFW accuracy decreased with anhydramnios, although there was not a significant difference in the number of measurement with absolute difference < 10%.

Blann and Prien [30] studied a group of 162 women and compared ultrasonographic and clinical EFW before and after amniotomy in laboring women with actual birth weights. They found a significant difference between EFW variance, with − 2.5% before amniotomy and − 10.5% afterward. Pre-amniotomy AC had the strongest correlation with actual BW, explained by the fact that soft tissue measurement is more likely to be affected by changes in AFV. Of note, they used the Shepard [6] formula, which did not rank among the top ten performing formulas in our study. Additionally, this study was performed while patients were in labor, and it is unclear how a contracting uterus may factor in and affect ultrasound measurements.

Another study [31] examined a large cohort of 820 pregnancies between 22 and 42 weeks of gestational age, and analyzed nine different factors that may affect precision of EFW. These factors included gestational age, maternal BMI, fetal gender, fetal weight, amniotic fluid index, fetal presentation, placental location, time interval between estimation and delivery, and experts vs. less experienced operator. They found that the only factor that had a statistically significant impact was that of time interval between estimation and delivery, with an interval of greater than 7 days having a negative impact on estimation. Similarly, other studies also have not shown that amniotic fluid volume plays a pivotal role in EFW [29, 32]. Our findings are in accordance with those studies, as we found that the accuracy of the commonly used Hadlock IV formula was not affected by low amniotic fluid volume.

Our findings have several clinical implications. Some practitioners may choose to change the current formula they are using use in the setting of PPROM, as it ranks low among all other formulas. Others may be satisfied to realize that the formulas used by them today performs well enough for their purposes. Our findings also provide reassurance for practitioners using the Hadlock IV, regardless of fetal gender or level of amniotic fluid.

EFW by an ultrasound poses many research questions including choice of formula, customized versus population growth charts, growth velocity, factors impacting EFW, and more. While our study sheds light on the performance of EFW formulas in a specific setting, additional research is needed to better characterize our use of EFW.

Our study is not free from limitations, mainly its retrospective design. As a result, pertinent information, including race and body-mass index, were not available. As it was performed in a single institution this may affect the external validity. As well, although our mean interval between sonogram to delivery was 30 days, we included all patients with sonograms up to 14 days from the delivery. Nonetheless, most measurements were done within the seven-day interval from birth, and as can be seen from our results, most formulas performed quite well. Additionally, the size of our cohort and standard management protocol considerably strengthen the findings.

## Conclusions

In women with singleton gestation and PPROM, the Ott [12] formula for EFW was the most accurate, yet all of the top ten ranking formulas performed quite well. The commonly used Hadlock IV performed quite similarly to Ott’s formula, and is acceptable to use in this specific setting.

## Supplementary Information

**Additional file 1.**

## Data Availability

The datasets used and/or analyzed during the current study are available from the corresponding author on reasonable request.

## References

[1] Hadlock FP, Harrist RB, Sharman RS, Deter RL, Park SK (1985). Estimation of fetal weight with the use of head, body, and femur measurements--a prospective study. Am J Obstet Gynecol.

[2] Woo JS, Wan CW, Cho KM (1985). Computer-assisted evaluation of ultrasonic fetal weight prediction using multiple regression equations with and without the fetal femur length. J Ultrasound Med.

[3] Warsof SL, Gohari P, Berkowitz RL, Hobbins JC (1977). The estimation of fetal weight by computer-assisted analysis. Am J Obstet Gynecol.

[4] Warsof SL, Wolf P, Coulehan J, Queenan JT (1986). Comparison of fetal weight estimation formulas with and without head measurements. Obstet Gynecol.

[5] Vintzileos AM, Campbell WA, Rodis JF, Bors-Koefoed R, Nochimson DJ (1987). Fetal weight estimation formulas with head, abdominal, femur, and thigh circumference measurements. Am J Obstet Gynecol.

[6] Shepard MJ, Richards VA, Berkowitz RL, Warsof SL, Hobbins JC (1982). An evaluation of two equations for predicting fetal weight by ultrasound. Am J Obstet Gynecol.

[7] Jordaan HV (1983). Estimation of fetal weight by ultrasound. J Clin Ultrasound.

[8] Hadlock FP, Harrist RB, Carpenter RJ, Deter RL, Park SK (1984). Sonographic estimation of fetal weight. The value of femur length in addition to head and abdomen measurements. Radiology..

[9] Hsieh FJ, Chang FM, Huang HC, Lu CC, Ko TM, Chen HY (1987). Computer-assisted analysis for prediction of fetal weight by ultrasound-comparison of biparietal diameter (BPD), abdominal circumference (AC) and femur length (FL). Taiwan Yi Xue Hui Za Zhi.

[10] Shinozuka N, Okai T, Kohzuma S, Mukubo M, Shih CT, Maeda T (1987). Formulas for fetal weight estimation by ultrasound measurements based on neonatal specific gravities and volumes. Am J Obstet Gynecol.

[11] Combs CA, Jaekle RK, Rosenn B, Pope M, Miodovnik M, Siddiqi TA (1993). Sonographic estimation of fetal weight based on a model of fetal volume. Obstet Gynecol.

[12] Ott WJ, Doyle S, Flamm S (1986). Accurate ultrasonic estimation of fetal weight. Effect of head shape, growth patterns, and amniotic fluid volume. Am J Perinatol.

[13] Gabbay-Benziv R, Aviram A, Bardin R, Ashwal E, Melamed N, Hiersch L (2016). Prediction of small for gestational age: accuracy of different Sonographic fetal weight estimation formulas. Fetal Diagn Ther.

[14] Aviram A, Yogev Y, Ashwal E, Hiersch L, Hadar E, Gabbay-Benziv R (2017). Prediction of large for gestational age by various sonographic fetal weight estimation formulas-which should we use?. J Perinatol.

[15] Rosati P, Arduini M, Giri C, Guariglia L (2010). Ultrasonographic weight estimation in large for gestational age fetuses: a comparison of 17 sonographic formulas and four models algorithms. J Matern Fetal Neonatal Med.

[16] Aviram A, Yogev Y, Ashwal E, Hiersch L, Danon D, Hadar E (2017). Different formulas, different thresholds and different performance-the prediction of macrosomia by ultrasound. J Perinatol.

[17] Barel O, Maymon R, Barak U, Smorgick N, Tovbin J, Vaknin Z (2014). A search for the most accurate formula for sonographic weight estimation by fetal sex - a retrospective cohort study. Prenat Diagn.

[18] Villar J, Cheikh Ismail L, Victora CG, Ohuma EO, Bertino E, Altman DG (2014). International standards for newborn weight, length, and head circumference by gestational age and sex: the newborn cross-sectional study of the INTERGROWTH-21st project. Lancet..

[19] Dimassi K, Karoui A, Triki A, Gara MF (2016). Performance of ultrasound fetal weight estimation in twins. Tunis Med.

[20] Pils S, Springer S, Seemann R, Wehrmann V, Worda C, Ott J (2018). Reliability of sonographic fetal weight estimation in triplet pregnancies: a retrospective cohort study. Arch Gynecol Obstet.

[21] Tsakiridis I, Mamopoulos A, Chalkia-Prapa EM, Athanasiadis A, Dagklis T (2018). Preterm premature rupture of membranes: a review of 3 National Guidelines. Obstet Gynecol Surv.

[22] ACOG Practice Bulletin No. 188 (2018). Prelabor rupture of membranes. Obstet Gynecol.

[23] Duncan JR, Schenone C, Dorset KM, Goedecke PJ, Tobiasz AM, Meyer NL, et al. Estimated fetal weight accuracy in pregnancies with preterm prelabor rupture of membranes by the Hadlock method. J Matern Fetal Neonatal Med. 2020. p. 1–5. 10.1080/14767058.2020.1769593. 10.1080/14767058.2020.1769593 32441170

[24] Aviram A, Quaglietta P, Warshafsky C, Zaltz A, Weiner E, Melamed N (2020). Utility of ultrasound assessment in management of pregnancies with preterm prelabor rupture of membranes. Ultrasound Obstet Gynecol.

[25] Kramer MS, Platt RW, Wen SW, Joseph KS, Allen A, Abrahamowicz M (2001). A new and improved population-based Canadian reference for birth weight for gestational age. Pediatrics..

[26] Valea FA, Watson WJ, Seeds JW (1990). Accuracy of ultrasonic weight prediction in the fetus with preterm premature rupture of membranes. Obstet Gynecol.

[27] Rose BI, McCallum WD (1987). A simplified method for estimating fetal weight using ultrasound measurements. Obstet Gynecol.

[28] Toohey JS, Lewis DF, Harding JA, Crade M, Asrat T, Major CA (1991). Does amniotic fluid index affect the accuracy of estimated fetal weight in preterm premature rupture of membranes?. Am J Obstet Gynecol.

[29] Esin S, Hayran M, Tohma YA, Guden M, Alay I, Esinler D (2017). Estimation of fetal weight by ultrasonography after preterm premature rupture of membranes: comparison of different formulas. J Perinat Med.

[30] Blann DW, Prien SD (2000). Estimation of fetal weight before and after amniotomy in the laboring gravid woman. Am J Obstet Gynecol.

[31] Heer IM, Kumper C, Vogtle N, Muller-Egloff S, Dugas M, Strauss A (2008). Analysis of factors influencing the ultrasonic fetal weight estimation. Fetal Diagn Ther.

[32] Janas P, Radoń-Pokracka M, Nowak M, Staroń A, Wilczyńska G, Brzozowska M (2019). Effect of oligohydramnios on the accuracy of sonographic foetal weight estimation in at term pregnancies. Taiwan J Obstet Gynecol.

