# Supplementary material for: Comparison of sonographic fetal weight estimation formulas in patients with preterm premature rupture of membranes
Source: BMC Pregnancy Childbirth. 2021 Feb 19;21:149. doi: 10.1186/s12884-021-03631-w (PMC7893917; doi:10.1186/s12884-021-03631-w)
Supplement: Supplementary file 1 — Additional file 1. [file 12884_2021_3631_MOESM1_ESM.docx]

**Comparison of sonographic fetal weight estimation formulas in patients with preterm premature rupture of membranes**

Chelsie WARSHAFSKY MS, MD^1^

Stefania RONZONI MD, PhD^1^

Ms. Paula QUAGLIETTA^1^

Eran WEINER, MD^2^

Arthur ZALTZ, MD^1^

Jon BARRETT, MD^1^

Nir MELAMED, MD^1^

Amir AVIRAM, MD^1^

^1^ Sunnybrook Health Sciences Centre, Department of Obstetrics and Gynecology, Division of Maternal-Fetal Medicine, University of Toronto, Toronto, Ontario, Canada

^2^Edith Wolfson Medical Center, Department of Obstetrics and Gynecology, Division of Maternal-Fetal Medicine, Holon, Sackler Faculty of Medicine, Tel-Aviv University, Tel-Aviv, Israel

**Corresponding Author:**

Amir Aviram, MD

Division of Maternal-Fetal Medicine, Department of Obstetrics and Gynecology

Sunnybrook Health Sciences Centre

2075 Bayview Ave, Toronto, ON M4N 3M5

Tel: 647-915-4846

Email: amiraviram25@gmail.com

Table S1

| Formula | Reference | POE 10% for 1-7 days interval (n=442) | POE 10% for 8-14 days interval (n=123) |
| --- | --- | --- | --- |
| 1 | Hadlock 1 (1985) | 66.1% | 31.7% |
| 2 | Woo (1985) | 49.6% | 26.0% |
| 3 | Warsof (1986) | 52.5% | 43.9% |
| 4 | Vintzileos (1987) | 60.0% | 34.1% |
| 5 | Warsof (1977) | 38.7% | 10.6% |
| 6 | Shepard (1982) | 60.6% | 30.0% |
| 7 | Jordaan (1983) | 30.5% | 58.5% |
| 8 | Hadlock (1984) | 63.6% | 30.9% |
| 9 | Woo (1985) | 35.8% | 9.8% |
| 10 | Hsieh (1987) | 0.9% | 0% |
| 11 | Hadlock (1984) | 65.4% | 26.0% |
| 12 | Jordaan (1983) | 32.1% | 57.7% |
| 13 | Jordaan (1983) | 13.0% | 42.2% |
| 14 | Hadlock 2 (1985) | 66.7% | 25.2% |
| 15 | Woo (1985) | 49.8% | 13.0% |
| 16 | Shinozuka (1987) | 65.2% | 45.5% |
| 17 | Hsieh (1987) | 46.4% | 46.3% |
| 18 | Hadlock 3 (1985) | 67.0% | 22.7% |
| 19 | Combs (1993) | 61.8% | 48.7% |
| 20 | Ott (1986) | 68.8% | 41.4% |
| 21 | Hadlock 4 (1985) | 65.8% | 22.7% |
